# Bone Remodeling: Histone Modifications as Fate Determinants of Bone Cell Differentiation

**DOI:** 10.3390/ijms20133147

**Published:** 2019-06-27

**Authors:** Sun-Ju Yi, Hyerim Lee, Jisu Lee, Kyubin Lee, Junil Kim, Yeojin Kim, Jae-Il Park, Kyunghwan Kim

**Affiliations:** 1 School of Biological Sciences, College of Natural Sciences, Chungbuk National University, Cheongju, Chungbuk 361-763, Korea; 2 Korea Basic Science Institute, Gwangju Center at Chonnam National University, Gwangju 500-757, Korea

**Keywords:** bone differentiation, histone modification, acetylation, methylation, cleavage, osteoclast, osteoblast

## Abstract

The bone tissue is a dynamic complex that constitutes of several interdependent systems and is continuously remodeled through the concerted actions of bone cells. Osteoblasts are mononucleated cells, derived from mesenchymal stem cells, responsible for bone formation. Osteoclasts are large multinucleated cells that differentiate from hematopoietic progenitors of the myeloid lineage and are responsible for bone resorption. The lineage-specific differentiation of bone cells requires an epigenetic regulation of gene expressions involving chromatin dynamics. The key step for understanding gene regulatory networks during bone cell development lies in characterizing the chromatin modifying enzymes responsible for reorganizing and potentiating particular chromatin structure. This review covers the histone-modifying enzymes involved in bone development, discusses the impact of enzymes on gene expression, and provides future directions and clinical significance in this area.

## 1. Introduction

Individual lifestyle and environmental factors can directly interact with the genome to influence epigenetic alterations. These alterations may exert their effects at different stages in a person’s life and even in later generations. Epigenetics is the study of heritable changes in gene expression in response to both internal and external environmental stimulation in the absence of alteration of underlying DNA sequence [1,2]. The major epigenetic mechanisms include histone modification, DNA methylation, and non-coding RNAs [3]. In particular, histone modification controls gene expression by influencing chromatin compaction or signaling other protein complexes [4]. Therefore, an appropriate balance of stability and dynamics in histone modification is necessary for accurate gene expression. Histones undergo diverse modifications including methylation, acetylation, propionylation, butylation, crotonylation, 2-hydroxyisobutylation, malonylation, succinylation, formylation, ubiquitination, citrullination, phosphorylation, *O*-GlcNAcylation, and ADP ribosylation [4,5,6,7,8,9,10,11,12]. Histone modifications exert effects on chromatin remodeling to reorganize the chromatin structure to expose or hide regions of DNA for gene expression. Histone modifications may cause changes in interaction of different histones or histone–DNA interaction [6,13]. Moreover, distinct patterns of histone modifications called “histone codes” present signals for recruiting specific protein complexes, including transcription factors or proteins responsible for the modification of chromatin structure [11,14,15]. A typical histone modification mechanism is the selective addition or removal of specific histone modification by histone-modifying enzymes. An alternative mechanism is the histone tail cleavage, which removes pre-existing tail modifications, but maintains the core histone region [16,17,18,19]. Several studies have demonstrated that epigenetic regulations play a pivotal role in bone cell development.

The bone is a rigid organ that supports body structure, protects our vital organs, provides an environment for bone marrow, and stores minerals such as calcium and phosphates. The bone is also a highly dynamic organ that undergoes remodeling throughout life to maintain bone strength and mineral homeostasis [20]. Bone remodeling is carefully coordinated by bone-resorbing osteoclasts and bone-forming osteoblasts. An imbalance between bone resorption and bone formation can cause bone diseases. Excessive bone resorption results in bone loss and osteoporosis, while osteopetrosis results from excessive bone formation [21].

**Figure 1:**
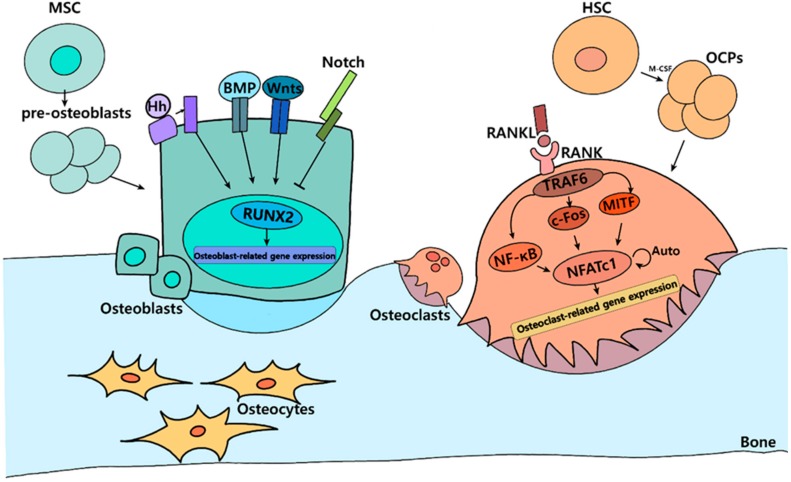
Signaling pathways of bone remodeling. At a specific area of damaged or old bone, osteocytes recruit osteoclast precursor cells (OCPs), which are differentiated from hematopoietic stem cells (HSCs). The receptor activator of nuclear factor kappa-B ligand (RANKL) binds to RANK on OCPs membrane and activates multiple signaling pathways, inducing nuclear factor of activated T-cells, cytoplasmic 1 (NFATc1) via nuclear factor-kappaB (NF-κB), c-Fos, or microphthalmia-associated transcription factor (MITF). NFATc1, as a master transcription factor, stimulates osteoclastogenic genes and thus osteoclast differentiation. Mature osteoclasts resorb a damaged or old bone matrix and subsequently osteoblasts are recruited into resorbed bone surfaces and from new bone. Mesenchymal stem cells (MSCs) differentiate into osteoblasts through various signaling pathways, such as bone morphogenic protein (BMP), Wnt, Hedgehog (Hh), and Notch. Runt-related transcription factor 2 (RUNX2) is a primary transcription factor, implicated in those pathways as a focal point for signaling integration.

Osteoblasts are derived from mesenchymal stem cells (MSCs), which play a critical role in bone formation. Osteoblasts are cuboidal cells harboring abundant rough endoplasmic reticulum and prominent Golgi apparatus, as well as various secretory vesicles. Osteoblasts can also differentiate into osteocytes, which are stellate cells populating the narrow interconnecting passages within the bone matrix. The expression of runt-related transcription factors 2 (RUNX2), distal-less homeobox 5, and osterix (OSX) is critical for osteoblast differentiation. In particular, RUNX2 functions as a primary transcription factor and is involved in proliferation, migration, commitment, and differentiation to the osteogenic lineage [22,23]. The Wnt/Notch system, Sox9, Msx2, bone morphogenetic protein (BMP), and hedgehog signaling are found upstream of RUNX2 [24,25]. RUNX2 plays an essential role in expressing osteoblast-related genes such as alkaline phosphatase, collagenase 1, osteocalcin (OCN), bone sialoprotein (BSP), and osteopontin (OPN) (Figure 1) [20].

Osteoclasts are large multinucleated cells with ruffled membranes, formed by the fusion of mononuclear precursors, and responsible for bone resorption and degradation [26]. Osteoclasts are differentiated from mononuclear cells of the hematopoietic stem cell lineage. Osteoclast precursor cells of monocyte-macrophage lineage fuse to form tartrate-resistant acid phosphatase (TRAP)-positive multinucleated cells. This differentiation is stimulated by several factors such as macrophage colony-stimulating factor (M-CSF) and receptor activator of nuclear factor kappa-B ligand (RANKL) [27]. Osteoblast-derived M-CSF binds to its receptor (CSF receptor 1, cFMS) in osteoclast precursors, which stimulates the proliferation and differentiation of osteoclast precursors and is also required for the survival, motility, and spread of osteoclasts [28]. RANKL is indispensable for the formation, fusion, activation, and survival of osteoclasts [27]. RANKL is secreted by osteoblasts, osteocytes, and stromal cells and binds to its receptor RANK on osteoclasts and its precursors. The binding of RANKL to RANK receptor results in the recruitment of tumor necrosis factor receptor-associated factor 6 (TRAF6), which in turn activates diverse signaling pathways such as nuclear factor kappa B (NF-κB), c-JUN N-terminal kinase (JNK), p38 mitogen-activated protein kinase, and extracellular signal-related kinase pathways [29,30]. The nuclear factor-activated T cells c1 (NFATc1), a master regulator of osteoclastogenesis, cooperates with microphthalmia-associated transcription factor (MITF), c-FOS, and NF-κB, regulating diverse osteoclast-related genes such as TRAP, cathepsin K, osteoclast-associated receptor, and matrix metalloproteinase-9 (MMP-9) (Figure 1).

The differentiation and function of osteoblasts and osteoclasts require regulated expression of different genes and coordinated actions of transcription factors along with coactivators and corepressors to regulate appropriate activation or repression of gene expression [26,31]. An increasing number of studies have defined the epigenetic mechanism as the coordinated and orderly regulation of gene expression in bone remodeling [26,31,32,33]. Recently, a great deal of progress has been made in exploring the changes in global histone modifications during osteoblastogenesis or osteoclastogenesis.

In this review, we provide a broad overview of histone modifications during osteoblastogenesis and osteocalstogenesis. We discuss the regulatory roles of histone-modifying enzymes and focus on the most extensively studied histone modifications, such as histone acetylation, histone methylation, and histone tail cleavage in osteoblastogenesis and osteoclastogenesis.

## 2. Histone Acetylation in Osteoblastogenesis and Osteoclastogenesis

### 2.1. Histone Acetylation

Histone acetylation occurs at the ε–amino group of lysine, largely in the histone tail. It is generally associated with transcription activation and open chromatin as acetylation removes positive charges from lysine residues on histones, leading to a weakening interaction between histone and DNA [6,13].

The representative acetylation sites include H3K9, H3K14, H3K18, H3K23, H4K5, H4K8, H4K12, and H4K16. Of the enzymes that catalyze modifications on histone, the enzymes that perform lysine acetylation (histone/lysine acetyltransferases, HATs/KATs) and deacetylation (histone/lysine deacetylases, HDACs/KDACs) were the first identified and have been extensively studied [34,35,36,37]. Generally, HATs are thought to function as coactivators in transcription as they catalyze lysine acetylation and loosen chromatin structure. On the contrary, HDACs are regarded as corepressors.

HATs can be divided into at least five different subfamilies based on structural and functional similarity of their catalytic domains, HAT domains: HAT1 (named histone acetyltransferase 1 as the founding member of the superfamily or KAT1), p300/CBP associated factor (PCAF)/Gcn5 (named for its founding member yeast Gcn5 and its ortholog, PCAF, or KAT2A/KAT2B), MYST (named for the founding members MOZ, Ybf2/Sas3, Sas2, and Tip60, or KAT5), p300/CBP (named for the two human paralogs p300 and CBP, or KAT3B/KAT3A), and Rtt109 (named for its initial identification as a regulator of Ty1 transposition gene product 109, or KAT11) [38]. On the other hand, HDACs comprise four classical classes based on sequence similarity: the Class I Rpd3-like proteins (HDAC1, HDAC2, HDAC3, and HDAC8), the Class II Hda1-like proteins (HDAC4, HDAC5, HDAC6, HDAC7, and HDAC9), the Class III Sir2-like proteins (SIRT1, SIRT2, SIRT3, SIRT4, SIRT5, SIRT6, and SIRT7), and the Class IV protein (HDAC11) [39]. The Class I, II, and IV HDACs contain a classical deacetylase domain while the Class III contains a NAD+-dependent catalytic domain.

### 2.2. Role of Histone Acetyltransferase in Osteoblastogenesis and Osteoclastogenesis

Histone acetylation neutralizes the positive charge of lysine residues and allows the transcription factors to access the DNA. Many studies have demonstrated the recruitment of histone acetyltransferases to promoters of osteoblastic genes and linking to transcriptional activation (Table 1). Chromatin immunoprecipitations techniques showed that CBP and p300 are localized to promoters of osteoblastic genes during osteoblast differentiation [40,41,42,43,44,45,46]. Kim et al. reported that Vitamin D receptor recruited CBP and p300 to promoters of *Cyp* and *Opn* in response to 1, 25-dihydroxyvitamin D3, leading to histone H4 acetylation in intact osteoblasts [44].

P300/CBP-associated factor (PCAF/KAT2B) is another HAT involved in osteogenic differentiation. Several studies have reported that PCAF, in addition to p300 and RUNX2, is required for parathyroid hormone (PTH) activation of *MMP-13* transcription. PCAF was increasingly recruited to the *MMP-13* proximal promoter region after PTH treatment, facilitating an increase in RNA polymerase II recruitment and histone acetylation. The co-recruitment of p300 and PCAF played an important role in PTH stimulation of MMP-13 promoter activity [45,47]. Zhang et al. reported that PCAF is also implicated in the osteogenic commitment of MSCs. They showed that the expression of PCAF was significantly increased after osteogenic induction through Smad signaling, which stimulated the expression of BMP pathway genes by increasing histone H3K9 acetylation [48]. In addition, other histone acetyltransferases, monocytic leukemia zinc finger protein (MOZ/KAT6A), and MOZ-related factor (MORF/KAT6B) physically and functionally interact with RUNX2, suggesting that they are involved in osteogenic differentiation [49].

Recent genome-wide studies characterized chromatin landscape in osteogenesis from MSCs [50,51]. Hakelien et al. found that osteoblast differentiation induced global enrichment of H3K4me3, H3K9ac, H3K27ac, and H3K36me3 marks, whereas a change of repressive methylation, H3K27me3, largely inversely correlate with changes of active marks [50]. Meyer et al. reported that distinct chromatin patterns in MSCs determine osteogenic and adipogenic differentiation. They found that active histone marks such as H3K9ac, H4K5ac, H3K4me1, H3K4me3, and H3K36me3 are essential for multipotent differentiation of MSCs [51].

HATs were also required for the expression of osteoclast-related genes. During osteoclastogenesis, p300 interacts with MITF, which plays a critical role in osteoclast differentiation, assuming that its acetyltransferase activity is involved in transcription activation [52]. It has been reported that CBP binds to promoters of genes that are critical for osteoclast differentiation [53,54,55]. Asagiri et al. showed that the selective auto-amplification of NFATc1 requires the association of NFATc1 and CBP to the *NFATc1* promoters. They revealed that the *NFATc1* promoter was increasingly associated with PCAF and CBP, concomitant with dissociation of histone deacetylase 1 (HDAC1) during osteoclastogenesis [53]. Recently, the possible role of histone acetyltransferase of the MYST family in osteoclastogenesis was presented by Meier et al. They demonstrated that a selective pharmacological inhibition of Bromodomain and PHD finger-containing protein strongly impaired RANKL-induced differentiation into bone-resorbing osteoclasts. Since the MYST family are recruited to chromatin by BRPF scaffolding proteins and bromodomain is a reader of acetylated lysine, these results suggested that the MYST family may be involved in osteoclast differentiation [56]. However, the role of the MYST family HATs in osteoclast differentiation remains elusive.

Besides histone acetylation, HATs can also acetylate non-histone proteins such as activators, thereby affecting their stabilization and activity [57,58]. Several studies have shown that acetylation of transcription factors by histone acetyltransferases increases the stability of transcription factors, leading to augmented osteoblastogenesis or ostoclastogenesis. Lu et al. reported that osterix acetylation at K307 and K312 by CBP facilitated its transcriptional activity and stability, which is required for osteoblast differentiation [59]. Another group demonstrated that PCAF acetylated RUNX2 and increased its transcriptional activity, involving osteoblast differentiation [60]. In addition, PCAF played an important role in RANKL-induced osteoclastogenesis. Kim et al. reported that PCAF acetylated and stabilized NFACTc1 proteins probably by blocking ubiquitin-mediated proteasome degradation [61].

### 2.3. Role of Histone Deacetylase in Osteoblastogenesis and Osteoclastogenesis

Like HATs, HDACs can also regulate gene expression by deacetylating both histone and non-histone proteins. Unlike HATs, however, HDACs positively or negatively influence bone cell development in a context-dependent manner (Table 1). It was reported that Class I HDACs such as HDAC1, HDAC2, HDAC3, and HDAC8 suppressed the expression of osteoblast-related genes [62,63,64,65,66,67]. Lee et al. demonstrated that HDAC1 was recruited to the promoter regions of *OSX* and *OCN*, resulting in the inhibition of osteoblast differentiation [65]. HDAC 3 interacted with RUNX2 to repress the osteocalcin promoter, and subsequently inhibit osteoblast differentiation [67]. Recently, Fu et al. showed that HDAC8 suppressed osteoblast-related genes expression by removing the acetylation of histone H3K9, thus leading to decreased osteogenic differentiation of BMSCs [63].

Class II HDACs, HDAC4, and HDAC5, associated with Smad3 and RUNX2, affect bone-regulating gene transcription. They acted as corepressors for TGF-β/Smad3-mediated transcriptional repression of RUNX2 function in differentiating osteoblasts [68]. Another report demonstrated that HDAC4 and HDAC5 deacetylated RUNX2, allowing the protein to undergo Smurf-mediated degradation [69]. Jensen et al. has shown that HDAC7 associated with RUNX2 repressed its activity during osteoblast maturation in a deacetylation-independent manner [70]. In contrast to other HDACs, SIRT1, a member of Class III HDACs, promoted the differentiation of MSCs toward osteoblasts. The activation of SIRT1 by resveratrol facilitated osteogenesis of human MSCs by upregulating *RUNX2* gene expression via SIRT1/FOXO3A axis [71]. In addition, Simic et al. reported that SIRT1 deacetylated β-catenin to promote its accumulation in the nucleus, leading to transcriptional activation of osteoblast-related genes [72]. Recently, it was reported that SIRT3 enhances superoxide dismutase 2 (SOD2) activity through SOD2 deacetylation, which regulates mitochondrial stress and enhances osteoblastogenesis. Furthermore, Sirt3 −/− mice exhibited obvious osteopenia [73].

The Class I HDACs play various roles in osteoclast differentiation. The HDAC1 functions as a transcriptional corepressor by localizing the promoters of osteoclast genes [74,75], whereas HDAC2 and HDAC3 promoted osteoclast differentiation [76,77]. Generally, Class II and IV HDACs are known as negative regulators of osteoclastogenesis [77,78,79,80]. Knockdown of HDAC4, HDAC5, HDAC7, HDAC9, or HDAC10 resulted in increased osteoclastogenesis and expression of osteoclast genes. Kim et al. reported that HDAC5 negatively regulated osteoclast differentiation by destabilizing NFATc1 protein [61]. Like other HDACs, Class III HDACs acted as negative regulators of osteoclasts. Knockdown of Sirt1, Sirt3, and Sirt6 promoted osteoclast differentiation and resulted in an increase of osteoclast-specific genes [81,82,83,84,85]. Sirt1 activation attenuated osteoclastogenesis by deacetylating and, thereby, stimulating FoxO activity, an anti-osteogeneic effector [83]. Sirt3 controlled the protein stability of AMPK, leading to negative regulation of osteoclast differentiation [81]. However, it was reported that Sirt6 cooperates with Blimp1 to positively regulate osteoclast differentiation through transcriptional repression of MafB, a transcriptional factor. Moreover, Sirt6-deficient mice in osteoclast showed increased bone mass [86]. HDAC11, Class IV HDAC, was thought to repress osteoclastogenesis by regulating the expression of *Dc-stamp* and *Ctsk*, but not *NFATc1* [78]. Although the roles of HDACs are intensively studied during bone cell development, the study regarding the specific residues of modified histones and specific target promoters of osteoclast-related genes modulated by HDACs must be determined.

## 3. Histone Methylation in Osteoblastogenesis and Osteoclastogenesis

### 3.1. Histone Methylation

Histone methylation occurs on all basic amino acid residues—lysines, arginines, and histidines. The most extensively studied histone methylation sites include lysine 4 on histone H3 (H3K4), H3K9, H3K27, H3K36, H3K79, and H4K20. Sites of arginine (R) methylation include H3R2, H3R8, H3R17, H3R26, and H4R3. Moreover, many other residues on the histone proteins H1, H2A, H2B, H3, and H4 have been identified as methylated, using mass spectrometry and quantitative proteomics [87]. Histone methylation contributes to either active or repressive chromatin structure, depending on the histone and the residue of modification. Methylation of H3K9, H3K27, or H4K20 is often associated with repression of transcription, whereas methylation of H3K4, H3K36, H3K79, and H3R17 is largely associated with activation of transcription [15,88,89].

Histone methyltransferase catalyzes the addition of methyl groups to lysines or arginines on histones. They are divided into three groups: the SET domain-containing lysine methyltransferase, DOT1-like lysine methyltransferase, and the protein arginine N-methyltransferase (PRMT) family. The SET domain lysine methyltransferases include SET1 family (MLL, MLL4, MLL3, MLL2, SET1, SET1L), SUV39 family (SUV39H1, SUV39H2, EHMT2/G9, SETDB1), SET2 family (NSD1, WHSC1, ASH1L, SET2L, SETD2), EZH family (EZH1, EZH2), SMYD family (SMYD1-5), PRDM family (PRDM1-16), and other SET domain-containing families (SET7/9, SETD8). There are two types of PRMTs. Type I PRMTs (PRMT1, 3, 4, 6, and 8) asymmetrically dimethylate their arginines while Type II PRMTs (PRMT5, PRMT7) symmetrically dimethylate their arginines. They dimethylate specific arginines not only on histones, but also other various proteins such as heterogeneous nuclear ribonucleoproteins and splicing proteins and nucleolar and ribosomal proteins [87,90,91].

There are two conserved families of histone demethylases which use different reaction mechanisms: LSD demethylases and JMJC demethylases. The LSD family members, including LSD1 and LSD2, use a FAD-dependent amine oxidation reaction to demethylate their substrates. Lysine specific demethylase 1 (LSD1) (also known as KDM1A) is the first identified histone demethylase, which catalyzes demethylation of H3K4me1, H3K4me2, H3K9me1, and H3K9me2 [92,93]. The second family is JmjC domain-containing histone demethylase family, which are 2-oxoglutarate-Fe(II)-dependent dioxygenases, removing methyl groups from lysine residues [91]. The human genome contains approximately 30 JmjC domain-containing proteins, and until now, 18 of those proteins have been shown to possess histone demethylase [92].

### 3.2. Role of Histone Methyltransferase in Osteoblastogenesis and Osteoclastogenesis

A growing body of evidence demonstrated that histone methyltransferase is involved in bone cell differentiation (Table 2). For example, Wolf–Hirschhorn syndrome candidate 1 (WHSC1), also known as NSD2, is a histone H3 lysine 36 (H3K36) tri-methyltransferase which increases the interaction of RUNX2 and p300, leading to activation of bone-related genes via H3K36 trimethylation [94]. Moreover, it was observed that H3K36me3 catalyzed by SETD2 regulates the fate of bone marrow MSCs. H3K36me3 decreased accordingly with an increase in SETD*2* level during osteoblast differentiation. SETD*2* depletion decreased osteoblast differentiation and SETD*2*-deficient mice showed declined bone mass. SETD2-mediated H3K36me3 regulates transcriptional initiation and elongation of the *Lbp* gene [95].

Enhancer of zeste homolog 2 (EZH2/KMT6) has been reported to suppress osteoblastogenesis via H3K27me3 methylation on the promoters of osteoblast-related genes [96,97,98]. EZH2 is also involved in age-dependent MSC differentiation into osteoblasts [99]. Dudakovic et al. demonstrated that EZH2 performs a bifunctional role during bone formation by suppressing the osteoblast-related genes while simultaneously inducing proliferative expansion of osteoprogenitor cells [100]. Collectively, these results suggested that EZH2 functions as a suppressor of MSC osteogenic differentiation, through the regulation of key osteogenic genes, *RUNX2*, *OP*, *OC*, *ZBTB16*, *MX1*, and *FHL-1* through H3K27me3 on their promoters [96,101]. Histone methyltransferase SUV420H2 was also known to play an important role in osteoblast differentiation. Analysis on the loss of function of SUV420H2 using siRNA demonstrated the global loss of H4K20 methylation and decreased expression of bone biomarkers and osteogenic transcription factors. Furthermore, SUV420H2 was required for matrix mineralization during osteoblast differentiation [102].

PRMT5 inhibition promoted osteogenic differentiation of mesenchymal stromal cells and blocked global symmetric dimethylation of H3R8 and H4R3, but not of H3R2. These results indicate that PRMT5 controls the differentiation potential of MSCs during osteogenic differentiation [103]. Recently, PRMT1 has been described to be required for osteoclastogenesis. PRMT1 regulated the transcriptional activity of p65 by direct interaction with p65 in RANKL-mediated osteoclastogenesis. It was observed that osteoclast differentiation was blocked in PRMT1 knockdown or PRMT1+/− BMMs compared to PRMT1+/+ BMMs. In addition, PRMT1 haploinsufficiency reduced the enzyme activity of TRAP and increased the BMD in ovariectomized mice [104]. It was reported that methyltransferase G9a regulates osteogenesis via *Twist* gene repression. Loss of G9a activity in conditional knockout mice leads to histone demethylation of H3K9me2 and subsequent prolonged activation of *Twist* genes, transiently repressing *Runx2* gene and the osteogenic program [105]. Recently, our research group found that H3 monomethylation at lysine 27 by G9a is essential for the enzymatic activity of MMP-9 and facilitates osteoclast differentiation [106]. Fang et al. showed that EZH2 promotes osteoclastogenesis by downregulating IRF8, a negative regulator of osteoclastogenesis. They found that RANKL stimulates the recruitment of EZH2 to *IRF8* promoter, leading to the deposition of the repressive H3K27me3 histone mark and the decrease in *IRF8* expression [107]. Gao et al. demonstrated that DOT1L, a histone methyltransferase of H3K79me, suppressed osteoclast differentiation. They revealed that DOT1L-mediated H3K79 methylation is mainly linked to osteoclast differentiation [108].

### 3.3. Role of Histone Demethylase in Osteoblastogenesis and Osteoclastogenesis

Several studies have evaluated the role of demethylases in osteoblast commitment and differentiation (Table 2). MSCs are multipotent stromal cells that have a great capacity for self-renewal while differentiating into osteoblasts, chondrocytes, myocytes, or adipocytes. Wang et al. reported that histone demethylase KDM4B and KDM6B promote osteogenic differentiation of human MSCs. The knockdown of KDM4B or KDM6B blocked osteoblastogenesis, but increased adipogenesis from MSCs. KDM4B enhanced *DLX* expression by removing H3K9me3, and KDM6B increased *HOX* expression by removing H3K27me3. The *DLX* gene and *HOX* gene are involved in osteoblast differentiation [109]. The expression of *Kdm6b* (*Jmjd3*) was induced during osteoblastogenesis in MC3T3-E1 cells. Knockdown of *Kdm6b* blocked osteoblast differentiation and bone formation and suppressed the expression of *Runx2*, *Osx*, *Opn*, *Bsp*, and *Ocn*. It was found that knockdown of *Kdm6b* elevated the level of H3K27me3 on the promoter of *Runx2* and *Osx*. These results show that KDM6B plays important roles in osteoblast differentiation [110].

KDM6A (UTX) and EZH2 have also been shown to function as an epigenetic switch to regulate mesenchymal stem cell lineage specification [96,111]. The transcript level of *Kdm6a* increased, but the transcript level of *Ezh2* gene decreased under osteogenic differentiation conditions. Conversely, under adipogenic differentiation conditions, the transcript level of *Kdm6a* decreased, but the transcripts level of *Ezh2* increased. This study concluded that KDM6A promoted osteoblast differentiation and inhibited adipogenesis, regulating H3K27me3 on the promoters of master regulatory genes. JARID1B/KDM5B was reported to play an important role in osteoblast-lineage commitment. JARID1B demethylated H3K4me3 on P1 promoter of *RUNX2*, suppressing the differentiation to osteoblatic lineage [112]. NO66, a JmjC containing protein, interacted with osterix and inhibited the activation of osterix-mediated promoter. NO66 exhibited histone demethylation activity specifically for H3K4me3 and H3K36me3, which was required for the regulation of osteoblast-specific promoter genes, such as *Bsp* or *Ocn* [113,114,115]. Recently, LSD1 was found to inhibit the differentiation of human MSCs toward osteoblasts in vitro. The deletion of LSD1 in osteoblast progenitor cells leads to increased bone mass. LSD1 negatively regulated the expression of *BMP2* and *WNT7B* via demethylation of H3K4me2 on promoter [116].

Yasui et al. reported that extreme reduction of H3K27me3 was observed around transcription start sites (TSSs) of *NFAT1c1* gene without alteration of the levels of H3K4me3 in RANKL-induced osteoclastogenesis [117]. They examined the involvement of H3K27me3 demethylases, such as JMJD3 and UTX (KDM6A), in the reduction of H3K27me3 after RANKL treatment. *Jmjd3* mRNA expression was elevated, but the level of *Utx* expression did not change significantly. They demonstrated that *Jmjd3* knockdown accumulated H3K27me3 around the TSSs of *Nfatc1* gene locus and blocked osteoclastogenesis, while *Utx* knockdown had no effect on osteoclastogenesis [117]. JMJD7 was found to act as a negative regulator of osteoclastogenesis. The level of *Jmjd7* mRNA and the occupancy of JMJD7 at the promoter regions of osteoclast-related genes were significantly downregulated during differentiation of osteoclast. Knockdown of *Jmjd7* enhanced the differentiation of osteoclast, the expression of osteoclast-related genes, such as *c-Fos*, *Dc-stamp*, *Ctsk*, *Acp5*, and *Nfatc1*, and the bone resorptive functions of the [118]. However, molecular mechanisms were not fully investigated in this study.

In addition, histone demethylases exert their effect on non-histone proteins, controlling osteoblastogenesis. PHF2, known as a JmjC containing protein which demethylases H3K9me2, promoted osteoblast formation, enhancing the DNA binding activity of RUNX2. Notably, it was found that the increase of osteoblastogenesis is dependent on the demethylation of RUNX2, not H3K9me2 [119]. Youn et al. demonstrated that JMJD5 negatively regulated osteoclastogenesis [120]. They found that JMJD5 hydroxylated NFATc1, leading to NFATc1 degradation. JMJD5 knockdown accelerated osteoclast formation and increased the expression of osteoclastogenesis-related genes such as *Ctsk*, *Dc-stamp*, and *Acp5*.

## 4. Histone Cleavage in Osteoclastogenesis

### 4.1. Histone Cleavage

Reversible histone modifications can be removed by histone-modifying enzymes. Recently, histone N-terminal tail cleavage (proteolysis) has become an interesting mechanism that removes pre-existing multiple modifications physically and irreversibly. A significant number of studies have demonstrated that the histone tail cleavage regulated a variety of cellular responses, including stem cell differentiation, osteoclast differentiation, granulocyte differentiation, mammary gland differentiation, aging, and DNA damage response. Numerous specific proteases are identified in various cellular processes related to histone cleavages, such as tryptase, MMP-9, cathepsin L, cathepsin D, glutamate dehydrogenase, JMJD5, and JMJD7 [54,121,122,123,124,125,126,127,128].

### 4.2. Role of Histone Cleavage in Osteoclastogenesis

**Figure 2:**
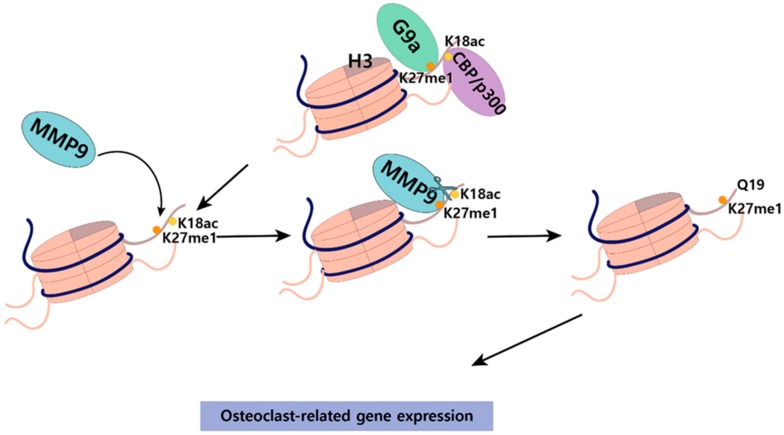
Schematic model of histone H3 N-terminal cleavage regulating osteoclastogenic target gene expression. G9a-mediated H3K27me1 at histone H3 enables matrix metalloproteinase-9 (MMP-9) to localize at target genes. MMP-9 catalyzes histone H3 N-terminal proteolysis, leading to osteoclast-related gene expression. CBP/p300-mediated H3K18ac facilitates MMP-9 protease activity toward H3 N-terminal tail.

We found that the cleavage of histone H3 N-terminal tail was observed during osteoclastogenesis [54]. In this report, the nuclear localization and the cleaving activity of MMP-9 toward histone H3 is necessary for the transcription of osteoclastogenic genes. H3 N-terminal cleavage was selectively targeted near TSSs to a small group of osteoclast-related genes and the expression of most H3-cleaved genes was altered during osteoclastogenesis. Our follow-up experiments showed that MMP-9 enzymatic activity toward nucleosomal H3 N-terminus is dependent on p300-mediated H3K18ac and G9a-mediated H3K27me1, thus establishing important osteoclastogenic functions for the histone acetyltransferase, p300, and the histone methyltransferase, G9a (Figure 2) [106]. We also reported that the DNMT inhibitor 5-Aza-CdR significantly facilitated RANKL-induced osteoclast differentiation through CpG demethylation and transcriptional activation of *Mmp-9* gene. Contrarily, however, treatment of the HDAC inhibitor TSA suppressed osteoclast differentiation and elevated H3K27ac, leading to a decrease in H3K27me1 and, thus, MMP-9 localization at target genes [129].

Researcher groups have demonstrated that JMJD5 and JMJD7 are involved in osteoclast differentiation, although they did not elucidate their precise mechanism [118,120]. Interestingly, it was recently reported that JMJD5 and JMJD7 cleave histone N-terminal tails, modulating transcription regulation [123,127]. Therefore, the investigation of the possible role of histone tail cleavage by JMJD5 and JMJD7 in osteoclastogenesis may be of interest.

## 5. Conclusions and Future Perspectives

Over the years, many researchers have intensively studied the mechanisms underlying the osteoclast and osteoblast formation. Epigenetic regulation is mainly involved in bone cell development. During bone cell differentiation, epigenetic marks, including histone modification, are dynamically changed. Recent advances in epigenetics have helped us understand which histone-modifying enzyme(s) is involved in the regulation of bone cell development. In this review, we provide the accumulated information on the role of histone-modifying enzymes linked to their target proteins in osteoblastogenesis and osteoclastogenesis. In general, most HATs stimulate osteoblastogenesis and osteoclastogenesis, whereas HDACs inhibit the differentiation of osteoblast and osteoclast. On the contrary, histone methylation regulated by HMT and histone demethylase differentially modulates the bone cell differentiation in a context-dependent manner. Despite these advances, many questions remain unanswered on the mechanism of histone modifying enzymes. Although genetic studies using loss of function revealed the biological significance of histone-modifying enzymes in bone cell development, we need to extend our understanding around which enzymes write or erase specific modification on target proteins during differentiation. It is also important to understand the effects of their modification(s) on gene expression during bone development. Indeed, a better understanding of the epigenetic mechanisms of both normal and pathological bone metabolism help us to develop the innovative therapeutics in treating osteoporosis and other bone erosive diseases.

## Figures and Tables

**Table 1:** Role of histone acetyltransferase and deacetylase on bone cell development.

		Osteoblstogenesis				Osteoclastogenesis			
Name	Synonyms	Function	Histone Modifications Studied	Non-Histone Substrates or Interacting Proteins	Ref.	Function	Histone Modifications Studied	Non-Histone Substrates or Interacting Proteins	Ref.
Histone acetyltransferases									
PCAF	KAT2B	↑	H3K9ac	RUNX2 acetylation	[45,47,48,60]	↑		NFATc1 acetylation	[53,61]
P300	KAT3B	↑	H3K18ac, H3K16ac, H4ac		[40,41,43,44,45,46]	↑	H3K18ac	Interaction with MITF	[52]
CBP	KAT3A	↑	H4ac	Osterix acetylation at K307 and K312	[42,44,59]	↑	H3K18ac		[53,54,55]
MOZ	KAT6A, MYST3	↑		Interaction with RUNX2	[49]	↑			[56]
MORF	KAT6B, MYST4	↑		Interaction with RUNX2	[49]	↑			[56]
Histone deacetylases									
HDAC1		↓			[65]	↓			[74,75]
HDAC2		↓			[64,66]	↑			[76]
HDAC3		↓		Interaction with RUNX2	[62,67]	↑			[77]
HDAC4		↓		RUNX2 deacetylation; Interaction with SMAD3	[68,69]	↓			[78]
HDAC5		↓		RUNX2 deacetylation; Interaction with SMAD3	[68,69]	↓		NFATc1 deacetylation	[61,78]
HDAC7		↓		Interaction with RUNX2	[70]	↓			[77,80]
HDAC8		↓	H3K9ac		[63]				
HDAC9						↓			[78,79]
HDAC10						↓			[78]
HDAC11						↓			[78]
SIRT1		↑		Beta-catenin deacetylation	[71,72]	↓		FoxO deacetylation	[83,85]
SIRT3		↑		SOD deacetylation	[73]	↓			[81,82]
SIRT6						↓or↑		Interaction with Blimp1	[84,86]

↑, promotion of differentiation; ↓, suppression of differentiation.

**Table 2:** Role of histone methyltransferase and demethylase on bone cell development.

			Osteoblstogenesis			Osteoclastogenesis		
Name	Synonyms	Function	Histone Modifications Studied	Non-Histone Substrates or Interacting Proteins	Ref.	Function	Histone Modifications Studied	Ref.
Histone metyltransferases								
G9	EHMT2	↑	H3K9me2		[105]	↑	H3K9me1	[106]
WHSC1	NSD2	↑	H3K36me3		[94]			
SETD2		↑	H3K36me3		[95]			
EZH2		↓	H3K27me3		[96,97,98,99,100,101]	↑	H3K27me3	[107]
SUV420H2		↓	H4K20me3		[102]			
PRMT1		↑		Direct interaction with p65	[104]			
PRMT5		↓	H3R3me2s, H3R8me2s		[103]			
Dot1						↓	H3K79me2	[108]
Histone demetylases								
LSD1	KDM1A	↓	H3K4me2		[116]			
JMJD2B	KDM4B	↑	H3K9me3		[109]			
JARID1B	KDM5B	↓	H3K4me3		[112]			
UTX	KDM6A	↑	H3K27me3		[111]			
JMJD3	KDM6B	↑	H3K27me3		[109,110]	↑	H3K27me3	[117]
PHF2		↑		RUNX2 demetylation	[119]			
NO66		↓	H3K4me3, H3K36me3	Interaction with osterix	[113,114,115]			
JMJD5	KDM8					↓	(NFATc1 hydroxylation)	[120]
JMJD7						↓		[118]

↑, promotion of differentiation; ↓, suppression of differentiation.
